# Patient Bonding: Going Beyond Sitting and Standing

**DOI:** 10.1093/asj/sjad355

**Published:** 2023-12-12

**Authors:** Foad Nahai, Pauline Joy F Santos

“The good physician treats the disease; the great physician treats the patient who has the disease!”

—Sir William Osler^1^

The patient-physician relationship continues to evolve with the progress of technology and readily available medical information on social media and physician web sites, not to mention artificial intelligence. The savvy patient can comprehend disease processes and even research treatment options. Plastic surgeons are challenged by the ever-changing patient-physician relationship. Technology affords many benefits for both physicians and patients, but also risks distancing the patient from the doctor. How often have we seen our own physicians with a hand on the keyboard and one eye on the computer screen while taking our history or providing medical advice? Now, more than ever, we as aesthetic surgeons should limit and even restrict the intrusion of technology into our relationship with our patients. It is time to bond with our patients.

Patients present to the clinic asking for liposuction of their arms, not understanding that direct excision of skin and subcutaneous tissue may be needed for a good result. Some ask for a blepharoplasty when a brow lift may be more appropriate. It is our job and responsibility as aesthetic surgeons to educate our patients and foster a trusting relationship—a relationship in which the patient trusts us, and we trust our patient. This bonding and mutual trust is challenged by patient biases based on their surgeon's communication skills, both verbal and nonverbal. For some, patients’ social media may have influenced their impressions of the surgeon before they even meet in person. The advent of telemedicine has led the patient-physician relationship to become more nuanced.^2,3^ How do physicians relate to their patients? How has it changed? And why is it important to be self-critical about how we relate to our patients?

Plastic surgeons, especially those of us engaged in aesthetic surgery and cosmetic medicine, unlike most other physicians, connect with our patients in a unique way. We do not typically “save lives,” but we do improve quality of life. We provide a service, not a commodity, for our patients that improves self-esteem.^4,5^ But even when we achieve our patient's goals, if we have not bonded with our patients, there may be problems. There are some corporate plastic surgery practice models in which the patient does not even know their surgeon. However, most plastic surgeons meet their patients and develop a relationship. This humanistic aspect of medicine is intrinsic to our field. As physicians, we are privileged to learn things about our patients that they sometimes do not even share with their closest friends and family members. They entrust us with their health and care.

We strive to educate our patients that aesthetic procedures are not a commodity, but rather a service—a personal service. Dare we say we provide an experience for our patients? An experience that starts when the patient views the website and continues through the first visit with an opportunity to bond with the staff and surgeons. Just as no 2 surgeons have the same skills or the same hands, experience, or aesthetic vision for each patient, no 2 patients have the same aesthetic goals. It is through bonding and listening to the patient that both the surgeon and patient can decide if they are the right choice for each other.

Of the many approaches to enhancing the patient-physician relationship, the most effective is communication.^6^ Patients who perceive that the time spent with their physician was adequate report greater satisfaction.^6^ In a study on patients’ perception of time spent at the bedside, the effect of sitting vs standing was studied in postoperative spine inpatients.^6^ The doctor who sat instead of standing was perceived as spending more time in the room; moreover, this resulted in more patient satisfaction.^7^ The nonverbal communication we provide as physicians is often overlooked. Small changes can significantly impact how a patient feels or understands their disease process and the quality of the care provided by their physician. It is not the objective measure of time that matters, but rather the perception of time spent. The power of posture has been demonstrated in various clinical settings as well, but it cannot substitute for poor communication skills.^8^ An eye on the computer and a hand on the keyboard run contrary to these findings.

Beyond sitting and standing, how can we better bond with our patients? It depends on how well we know our patient. Being able to compliment a patient's outfit or asking a patient about their personal lives reflects the extent of rapport between physician and patient. Chatting with patients as we would with a friend allows the patient time to relax before we proceed to address clinical issues.^6^ Perhaps we can simply ask our patients what they enjoy spending time on, about their hobbies and interests. This not only demonstrates our interest in getting to know our patients, but also provides us with greater insight into how they spend their time.

It is a given that improved patient-physician communication enhances patient care. There is evidence that a more positive perception of one's physician decreases the likelihood of malpractice litigation in the event of an adverse outcome.^9^ We, as plastic surgeons, appreciate the value of bonding with our patients and connect with our patients in a unique manner. We improve quality of life through reconstructive and aesthetic surgery. As Sir William Osler so succinctly articulated, we treat the patient, not just the aesthetic or reconstructive need. Beyond providing competent care, bonding with our patients builds a foundation for a sustainable and long-term patient-physician relationship.
